# A Whey-Based Diet Can Ameliorate the Effects of LPS-Induced Growth Attenuation in Young Rats

**DOI:** 10.3390/nu15081823

**Published:** 2023-04-10

**Authors:** Chen Menahem, Michal Foist, Yasmin Mansour, Biana Shtaif, Meytal Bar-Maisels, Moshe Phillip, Galia Gat-Yablonski

**Affiliations:** 1 Sackler Faculty of Medicine, Tel Aviv University, Tel Aviv 6997801, Israel; 2 The Jesse Z. and Sara Lea Shafer Institute for Endocrinology and Diabetes, National Center for Childhood Diabetes, Schneider Children’s Medical Center of Israel, Petach Tikva 4920235, Israel; 3 Felsenstein Medical Research Center, Tel Aviv University, Petach Tikva 4920235, Israel

**Keywords:** LPS, inflammation, growth plate, soy, whey, hypertrophic zone

## Abstract

Chronic inflammation in childhood is associated with impaired growth. In the current study, a lipopolysaccharide (LPS) model of inflammation in young rats was used to study the efficacy of whey-based as compared to soy-based diets to ameliorate growth attenuation. Young rats were injected with LPS and fed normal chow or diets containing whey or soy as the sole protein source during treatment, or during the recovery period in a separate set of experiments. The body and spleen weight, food consumption, humerus length, and EGP height and structure were evaluated. Inflammatory markers in the spleen and markers of differentiation in the EGP were assessed using qPCR. The LPS led to a significant increase in the spleen weight and a decrease in the EGP height. Whey, but not soy, protected the animals from both effects. In the recovery model, whey led to increased EGP height at both 3 and 16 d post treatment. The most affected region in the EGP was the hypertrophic zone (HZ), which was significantly shortened by the LPS treatment but enlarged by whey. In conclusion, LPS affected the spleen weight and EGP height and had a specific effect on the HZ. Nutrition with whey protein appeared to protect the rats from the LPS-induced growth attenuation.

## 1. Introduction

Most chronic inflammatory diseases in childhood are characterized by impaired growth [1]. The mechanism underlying the pathophysiology of this process is not clearly understood but is known to be a complex phenomenon comprised of the chronic inflammation itself, suboptimal nutrition, and (in many cases) prolonged use of glucocorticoids. This makes managing pediatric inflammation a dual challenge for clinicians, because both the disease and the treatment affect growth [2].

Normal growth during childhood depends primarily on prenatal and perinatal health, genetics, adequate nutrition, a proper psychosocial environment, the absence of diseases, and a normal hormonal profile [3,4]. Short stature is not just a cosmetic issue but a rather major contributor to individuals’ general well-being, and it affects many facets of adult life. Because intervention in linear growth is only efficient during a specific time window in a child’s life, it is of paramount importance to take it into consideration in children and adolescents with inflammatory diseases and treat it accordingly.

There is ample research showing that standard growth hormone (GH) injection therapy does not have a significant effect on linear growth in children with inflammatory diseases [5]. Numerous studies have indicated that chronic inflammation can suppress the GH/insulin-like growth factor (IGF)-1 axis through several mechanisms, such as relative GH and/or IGF-1 insufficiency, peripheral resistance to GH and/or IGF-1 resulting from the downregulation of GH and IGF-1 receptors, disruption in the GH/IGF-1 signaling pathways, dysregulation of IGF-binding proteins (IGFBPs), reduced IGF bioavailability, and modified gene regulation due to changes in the microRNAs system [6,7]. In addition, pro-inflammatory cytokines [2], including tumor necrosis factor a (TNF- α), interleukin (IL) 1β, and IL-6, which were shown to be upregulated in inflammatory diseases, act directly on the epiphyseal growth plate (EGP) located at both ends of the long bones and inhibit their growth [2,7,8].

Linear growth in children depends on the concerted action of multiple hormones but primarily GH and IGF-1, thyroid hormones, insulin, and sex steroids. In addition, paracrine factors, extracellular matrix (ECM) molecules, and intracellular proteins act in concert to regulate chondrocytes in the EGP [4]. Endochondral ossification begins with the proliferation of early chondrocytes (in the reserve zone), followed by their alignment in columns (proliferation zone; PZ) and their maturation into hypertrophic chondrocytes (hypertrophic zone; HZ). The hypertrophic cells increase in volume by 5- to 10-fold, cease dividing, and boost the deposition of cartilage ECM components and the secretion of matrix vesicles that contain matrix-processing enzymes and serve as centers of mineralization. Chondrocyte hypertrophic differentiation is mediated by a reduction in collagen type II accompanied by an elevation in collagen type X (Coll X), which is regulated by the Runt-related transcription factor 2 (RUNX2). Thereafter, the chondrocytes undergo programmed cell death, with calcification of the ECM, or transdifferentiate into endochondral osteoblasts [9,10]. ECM remodeling is a critical rate-limiting step in endochondral bone formation. Several matrix metallopeptidases (MMPs) are involved in this remodeling, including MMP-13 (collagenase 3), an ECM-degrading enzyme that plays a crucial role in bone formation and remodeling due to its expression both in terminal hypertrophic chondrocytes in the EGP and in osteoblasts [11]. The cartilage scaffold is replaced by bone tissue in a process involving the vascular invasion of the EGP, resorption of the cartilaginous matrix by osteoclasts, and recruitment of osteoblasts that deposit the trabecular bone matrix. The chief biomarkers of chondrocyte hypertrophy are Coll X and MMP-13.

In this current study, we used the lipopolysaccharide (LPS) model of inflammation [12] and showed that it leads to reduced height of the EGP with a specific decrease in the HZ. LPS, a unique toxic structure on the cell wall of Gram-negative bacteria that reside in the intestines [13,14], can be released into the circulation and stimulate the immune system. It is frequently administered to animals in low concentrations as a model to create subclinical inflammation, as well as bone inflammation [12].

There is a general consensus that both healthy children and those suffering from chronic inflammation need optimal nutrition to support growth; the effects of malnutrition have been amply demonstrated in numerous studies in both animal models and children [15,16]. A growing body of evidence points to the positive effects of dairy products and particularly milk proteins on linear growth in older healthy children, and in those recovering from malnutrition [17]. At the same time, there is growing interest in plant-based foods for ecological and financial reasons. The production of plant-based foods requires less land and water, and it is associated with lower greenhouse gas emissions than animal-based foods. However plant-based proteins are considered to be lower in quality in terms of their ability to increase postprandial muscle protein synthesis rates [18] and linear growth, as exemplified by differences in male adult height [19]. In a previous study, we compared whey and soy in young rats. Both whey and soy proteins contain all the essential amino acids (EAA) and are considered the best proteins in their categories on the Protein Digestibility-Corrected Amino Acids Score (PDCAAS). We showed that whey was better for linear growth in healthy rats [20,21]. The current study extends these findings by exploring growth in young rats in an inflammatory model.

## 2. Methods

### 2.1. Animals

In all the experiments, we used young (21–24 d) male Sprague Dawley rats (purchased from Envigo Laboratories Ltd., Jerusalem, Israel). Because puberty starts later in males, they enable a longer intervention period. The experiments were conducted at the animal care facility of the Felsenstein Medical Research Center, according to the guidelines of the Institutional Animal Care and Use Committee of Tel Aviv University (Approval number 01-20-088). During the acclimation, dosing, and recovery phases, the animals were housed in conventional, polycarbonate cages with solid bottoms (3 per cage) with a plastic cylinder for shelter and comfort. Standard procedures and conditions were applied for animal care, feeding, and maintenance of room, caging, and the environment. The animals were fed the commercial 2018SC diet (normal chow, NC; containing proteins from a combination of grains, purchased from Envigo Laboratories Ltd., Jerusalem, Israel) ad libitum, with free access to filtered tap water. Automatically controlled environmental conditions were set to maintain temperature at 21 °C + 2 °C with 50% + 5% relative humidity, 12:12 h light: dark cycle, and lights off at 19:00 h. Based on a preliminary experiment to identify the best LPS dosing, inflammation was induced by administering 2 mg/kg/day of LPS (Sigma, St. Louis, MO, USA), at 16:00 h to match the rats waking hours, in line with the short half-life of LPS (7.5 h) [22]. Environmental conditions and clinical state were monitored daily. No difference in survival was noted between groups. Throughout this study, the animals were observed daily for changes in skin, fur, and behavior in response to handling. All remained within the normal limits for this model.

### 2.2. Feeding Regimens

**Figure 1:**
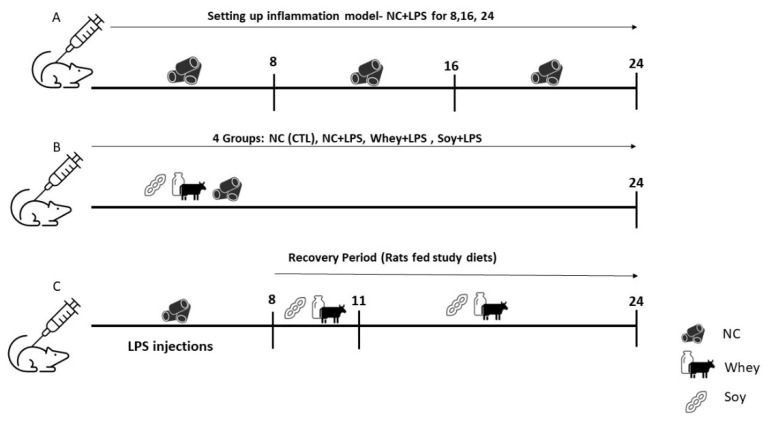
Study Design: Pilot study for the inflammation model. (**A**) Animals were given normal chow ad libitum for 8, 16, and 24 days and injected with LPS according to the assigned group (see methods). (**B**) Animals were randomized into 4 groups, injected with LPS (2mg/kg/day), and fed NC or the study diets for 24 days. (**C**) Animals were injected with LPS for 8 days and then fed the study diet for 3 or 16 days (recovery model).

The diets were iso-energetic and contained either soy protein (TD190912; Teklad, Envigo Diets, Madison, WI, USA) or whey (TD190911) as the sole protein source (Supplemental Table S1) [21]. All the other ingredients (corn starch, carbohydrate, cellulose, fat, vitamins, and minerals) were identical. The experimental design is depicted in Figure 1. The animals were given food ad libitum: NC (in Exp. 1; Figure 1A) or whey/soy diets when indicated (in Exps. 2 and 3; Figure 1B,C). Although the protein content in these enriched diets was higher than the standard NC (28% compared to 18% in NC), the protein consumption was similar (Supplemental Figure S1). In the recovery experiment (Exp. 3), after a period of 8 days on the LPS regime, the animals were randomly allocated to one of two groups, and LPS administration ceased. One was fed the whey diet and the other the soy diet for 3 or 16 days. All of the rats were euthanized by CO_2_ inhalation at the end of the experiments.

### 2.3. Growth Monitoring

In each experiment, food consumption and weight gain were measured throughout the experiment. Bone length and final weight were obtained at the end. At sacrifice, the blood and spleen were collected and frozen for later analysis. The humeri of the euthanized animals were carefully removed, cleaned, measured for length with a digital caliper, and fixed immediately for histo-morphometric analysis. EGP from the tibia was sterilely scraped and immediately flash-frozen in liquid nitrogen for further analysis.

### 2.4. Staining and Measurement of EGP Height

The humeri were fixed in 10% neutral buffered formalin for 48 h at room temperature, decalcified with Surgipath Decalcifier II (Leica Biosystems Richmond, Inc., Richmond, IL, USA) for several hours (depending on the age of the animal), dehydrated through a graded ethanol series (70%, 95%, and 100%), and stabilized by 2 sequential changes in chloroform for paraffin embedding. Histological studies and EGP height measurements were performed on deparaffinized 6 μm thick sections stained with hematoxylin-eosin and Alcian blue. For each bone, two sections were measured, for a total of five measurements per section. The height of the EGP was measured by drawing a straight line from the apical border of the reserve zone cells to the lower border of the mineralized cartilage. The proliferative and hypertrophic zones were measured separately from the apical border to the first hypertrophic cell and then from there to the lower border to the EGP. The slides were photographed under an Olympus BX40 microscope equipped with an Olympus DP71 camera (Olympus Optical Co. GmbH, Hamburg, Germany) and analyzed with Image-Pro software (version 4.5.1.22, Media Cybernetics, Inc., Rockville, MD, USA) by two specialists blind to their origin. The findings below represent the average of at least five measurements for each section.

### 2.5. Expression Analysis

RNA was extracted from the EGPs and the spleens using the RNeasy mini kit (Qiagen, Valencia, CA, USA). When extracting from the EGP, the isolated EGP was first homogenized on ice using a sterile scalpel, followed by homogenization with a pellet crusher and passing through a 21G needle and syringe. Ethanol precipitation was performed with 100% ethanol that was pre-chilled to −80 °C. First-strand cDNA synthesis was performed using the High Capacity RNA to cDNA Synthesis Kit (Intivrogen, Waltham, MA, USA) using 2 μg of the total RNA as a template, according to the manufacturer’s instructions. The real-time quantitative polymerase chain reaction (qPCR) was performed using the StepOnePlus™-RealTime PCR system (Thermo Fischer Scientific, Waltham, MA, USA), according to the manufacturer’s instructions using the primers in Table 1. The data were analyzed with GAPDH as the reference gene, using the standard 2^−ΔΔCt^ method.

### 2.6. Statistical Analysis

The data are presented as the mean ± standard deviation (SD). Kolmogorov–Smirnov test was used to test for the normality of the data. ANOVA with multiple comparisons or Student *t*-test were used to test for significant differences between experimental groups (indicated in the figure legends). Differences were considered statistically significant at *p* < 0.05.

## 3. Results

### 3.1. Pilot Study

**Figure 2:**
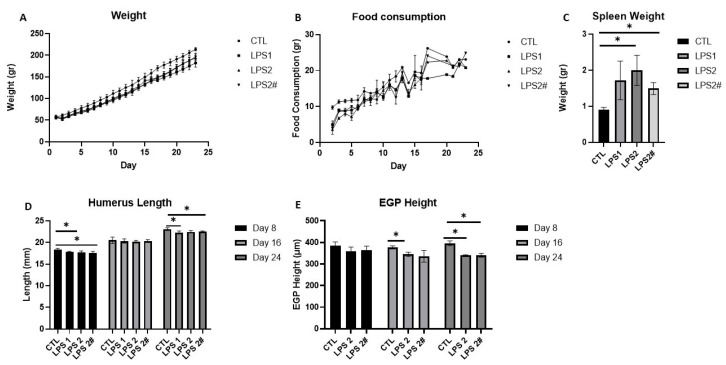
The effect of different doses of LPS (1 mg/kg/day, 2 mg/kg/day, and 2 mg/kg semi-daily *) on (**A**) weight, (**B**) food consumption, (**C**) spleen weight, (**D**) humerus length at 8, 16, and 24 days, (**E**) EGP height at 8, 16, and 24 days. Student’s *t*-test was used (control was compared to each dose); mean ± SD values are shown (n = 3 per group). LPS2 #—LPS 2 mg/kg semi-daily. * <0.05.

Because most studies on LPS have examined its effect on adult rats [12], we first needed to determine which LPS dose would be safe and effective in young rats. The rats were randomized into 4 groups (n = 36; n = 9 per group) and administered saline (CTL) or LPS injections (1 or 2 mg/kg) [23] every day or semi-daily (Figure 2) for 8, 16, and 24 days. The LPS was injected into the rats at 16:00 h, to match their waking hours. Weight gain and food consumption were measured throughout the experiment. At sacrifice, we measured the spleen weight, as a gross measure of inflammation, and the humerus length. As a proxy for the effect on linear growth, we measured the EGP height. The outcomes indicated that the best dose was 2 mg/kg/day because the combined effect on the spleen weight (Figure 2C) and EGP height (Figure 2E) was the most pronounced.

### 3.2. Effects of Soy and Whey on Linear Growth during Inflammation

**Figure 3:**
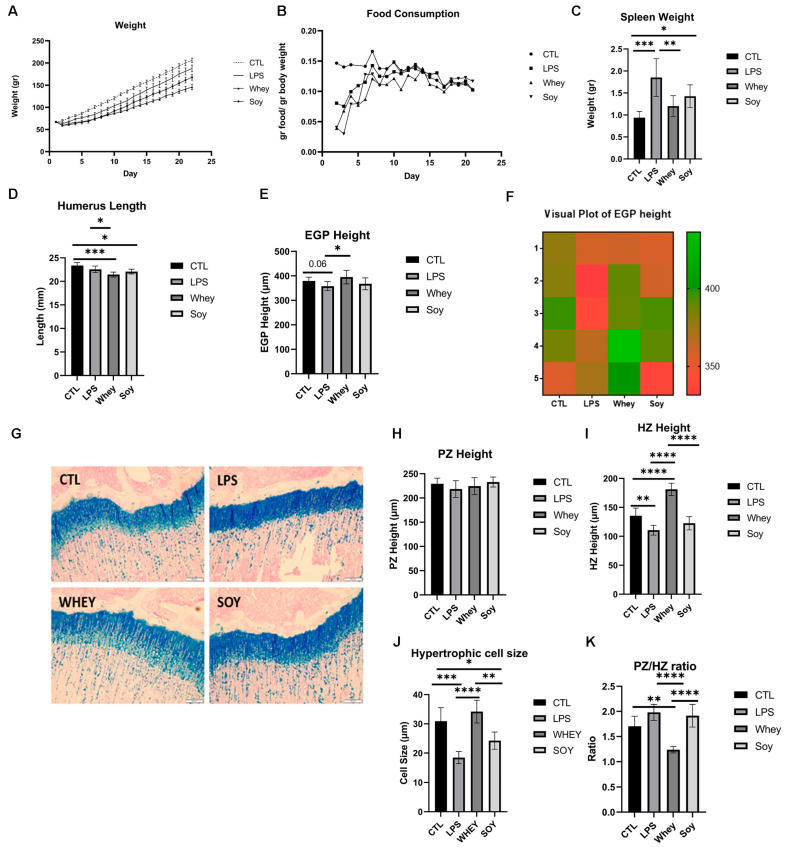
The effect of LPS and study diets on (**A**) weight, (**B**) food consumption, (**C**) spleen weight, (**D**) humerus length, (**E**) EGP height, (**F**) visual plot of EGP height, (**G**) representative stained sections of EGP from all groups, (**H**) PZ height, (**I**) HZ height, (**J**) hypertrophic cell size, (**K**) PZ/HZ ratio. ANOVA was used; (**A**–**E**,**H**–**K**) mean ± SD values are shown (n = 5/6 per group). * <0.05; ** <0.01; *** <0.001; **** <0.0001.

Next, we introduced whey or soy to the LPS-induced inflammation model and monitored the rats for 24 days. The animals treated with LPS had lower weights than the control rats (by 10%; *p* < 0.05; Figure 3A). An analysis of the effect of the diets showed that the whey-fed rats weighed the least throughout the experiment, although the normalized food consumption was equivalent in all groups (Figure 3B). As a surrogate marker for inflammation, we tested the spleen weight at sacrifice [24] (Figure 3C). The LPS-treated rats had a significantly larger spleen than the CTL group (the average spleen weight in the CTL group: 0.94 g ± 0.14; LPS: 1.85 g ± 0.42 (LPS compared to CTL, *p* = 0.0002)), as previously shown in mice [25]. A similar increase in the spleen weight was noted in the soy-fed group but not in the whey-fed group (LPS+Soy: 1.43 g ± 0.25 (compared to LPS, P = NS); LPS+Whey: 1.2 g ± 0.23 (compared to LPS, *p* = 0.007)). The humerus length was shorter in all the LPS-treated groups compared to the CTL (Figure 3D), with the whey and soy diets leading to a further shorter humerus length (soy by 5.5%, *p* = 0.01; whey by 9%, *p* = 0.0003). In contrast, while the EGP tended to be shorter in the LPS-treated rats (compared to the CTL, by 6%; *p* = 0.06), the whey protected the EGP from this effect; the height of the EGP was significantly larger than that of the LPS (by 10%, *p* = 0.03, LPS+whey compared to LPS). The height of the HZ that was reduced by the LPS (by 19%, *p* = 0.007 compared to the CTL) was increased in the whey-fed animals and surpassed all other groups (CTL 135 ± 11.7 μm; LPS 110 ± 7.7 μm; LPS+whey 181 ± 9.4 μm; LPS+soy 122 ± 10.2 μm; *p* < 0.0001 for all comparisons vs. LPS+whey; Figure 3I). The average number of hypertrophic cells was not affected; however, the average size was significantly lower in the LPS-treated animals (CTL 30.9 ± 4.1 μm; LPS 18.4 ± 1.9 μm; LPS+whey 34.2 ± 3.5 μm; LPS+soy 24.2 ± 2.6 μm; *p* < 0.05 for all comparisons apart from LPS+whey vs. CTL which was NS; Figure 3J). The ratio of the PZ to HZ was higher in the LPS and soy groups than in the CTL and whey-fed animals; the lowest ratio was observed in the whey group (*p* < 0.05 CTL vs. LPS+whey, all other comparison vs. LPS+whey *p* < 0.0001; Figure 3K). In all cases, the margins of the EGP were intact.

### 3.3. Analysis of Cytokine Expression in the Spleen

**Figure 4:**
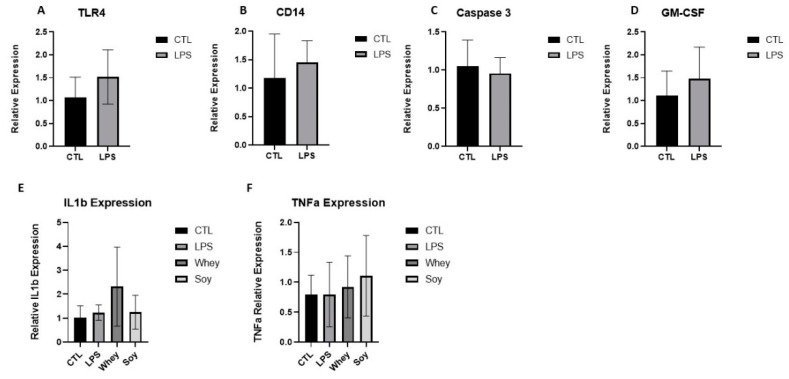
The effect of LPS and study diets on (**A**) TLR4, (**B**) CD14, (**C**) Caspase 3, (**D**) GM-CSF, (**E**) IL-1β, and (**F**) TNFα expression in rat spleens. (**A**–**D**) Student’s *t*-Test was used, (**E**,**F**) ANOVA was used. Mean ± SD values are shown.

Toll-like receptor 4 (TLR4) is an innate immune receptor for LPS. The binding of LPS activates a TLR4 signaling cascade and stimulates the PI3K/Akt pathway and nuclear factor κB (NF-κB) which triggers the subsequent biological effects [26]. We tested for multiple targets along this signaling pathway, including CD14, Caspase 3, and the Granulocyte-macrophage colony-stimulating factor (GM-CSF). LPS was also shown to induce the production of numerous cytokines, such as tumor necrosis factor α (TNF-α) and interleukin (IL) 1β, both of which have been shown to contribute to growth retardation [2,6,7]. However, we did not find any change in the LPS group or in the diet groups despite the significant changes in the spleen weight (Figure 4).

### 3.4. Effect of Whey vs. Soy on Linear Growth (Recovery Model)

**Figure 5:**
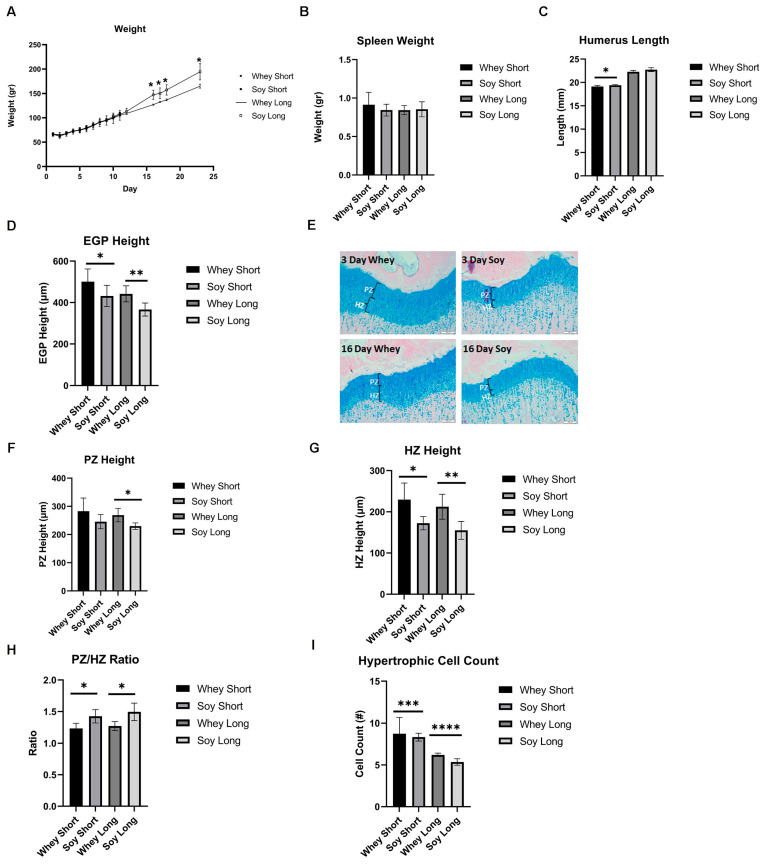
The effect of recovery from LPS-induced inflammation using study diets on (**A**) weight, (**B**) spleen weight, (**C**) humerus length, (**D**) EGP height, (**E**) representative stained sections of EGP from all groups, (**F**) PZ height, (**G**) HZ height, (**H**) PZ/HZ ratio, (**I**) hypertrophic cell count. Student’s *t*-test was used (the two time points were compared per each group). (**A**–**I**) Mean ± SD values are shown (n = 5/6 per group). * <0.05; ** <0.01; *** <0.001; **** <0.0001.

After 8 days of LPS injections, the treatment was discontinued, and the rats were fed either whey or soy and then were monitored for 3 or 16 days. During the recovery phase, there was a significant difference in weight gain, where the soy-fed animals gained more weight (at 16 days, *p* = 0.015) (Figure 5A). The effect of LPS on the spleen weight was no longer significant between the groups (Figure 5B). During the 3-day recovery phase, the soy-enriched diet led to a significantly longer humerus (although the difference was small (3%), *p* < 0.05); this effect diminished after 16 days (Figure 5C). The EGP height was greater in the whey-fed animals at both time points (3 days, *p* < 0.05; 16 days, *p* < 0.01; Figure 5D,E). The proliferative zone tended to be greater in the whey group after the 3-day recovery (*p* = 0.15) and the difference was significant after 16 days of feeding (*p* < 0.05; Figure 5F). The HZ was also greater in the whey-fed animals compared to the soy group in both the 3 and 16 days of recovery (at 3 days, *p* < 0.05; at 16 days, *p* < 0.01; Figure 5G), although the PZ/HZ ratio was greater in the soy group, as before (*p* < 0.05 at both time points; Figure 5H). In contrast to the previous model, the average number of hypertrophic cells was lower in the soy group than in the whey group at both time points (at 3 days, *p* = 0.0003; at 16 days, *p* < 0.0001; Figure 5I), but the calculated average size was not affected. In all cases, the margins of the EGP were intact, as before.

### 3.5. Analysis of Differentiation of EGP Chondrocytes

**Figure 6:**
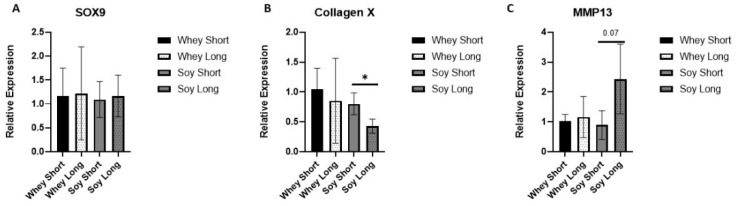
The effect of recovery from LPS-induced inflammation using the study diets on the relative expression of (**A**) SOX9, (**B**) collagen X, and (**C**) MMP13. Student’s *t*-test was used (two time points were compared per each group; * <0.05).

To further analyze the effects on the HZ, we tested three surrogate markers for hypertrophy differentiation: the SRY-Box transcription factor (SOX)-9, Coll X, and MMP-13 (Figure 6). The expression level of SOX9, a crucial gene modulating the differentiation of chondrocytes, was not affected. The Coll X expression declined significantly with time in the soy group (*p* < 0.05), whereas no such effect occurred in the whey group. In contradistinction, MMP-13 tended to increase in the soy EGP with time (*p* = 0.07) but not in the EGP of the whey-fed rats.

## 4. Discussion

The most important finding of this study is the differential effect of whey as compared to soy on inflammation (spleen weight), inflammation-induced growth attenuation, and its effect on the structure of the EGP. To the best of our knowledge, this is the first report on the effects of subclinical inflammation induced by LPS in young rats on linear growth, and specifically on EGP height and its internal organization. Importantly, the findings showed that the HZ was the most significantly affected. A recent study by Liu and colleagues suggested that idiopathic short stature (ISS) can be the result of subclinical inflammation, thus making this study even more relevant to children with growth attenuation, either due to bona fide chronic inflammatory disease or to subclinical inflammation [27]. Improving nutritional status is one way to improve linear growth in inflammatory conditions and ISS [28,29]. Although the positive effects of dairy products on linear growth are well-established, soy-based formulas are the formulas of choice for children with food allergies and for children whose parents opt to avoid food from animal-derived products for various reasons. This makes it all that more important to compare the effect of whey and soy on linear growth. Our conclusion that whey is better than casein or soy is based on our previous studies in which we showed that whey is keeping a larger EGP for a longer duration. In addition, we found that whey led to an increase in the level of IGF-1, better bone structure, and stimulated growth even in the presence of inflammation. Furthermore, whey led to a lower weight gain and as such may be important to circumvent the hazard of metabolic complications that are known to be a risk factor that accompanies catch-up growth [20,21,30].

LPS can be released from the gut into the circulation, where it stimulates the immune system. A 2023 meta-analysis by Bott et al. showed that LPS is a viable model for studying inflammation [12] and that systemically delivered exogenous LPS in rodents upregulates bone resorption [12]. Our study is one of the few that have used rats and not mice, and it is the only one to investigate its effects on the EGP.

In the current study, we showed that LPS increased the spleen weight. However, we did not find any change in the LPS or the diet groups in terms of the expression level of TLR4, CD14, Caspase 3, or GM-CSF. TLR4 is the receptor for LPS, and CD14 is a glycosyl-phosphatidyl-iositol-anchored plasma-membrane protein that associates physically with Gi/Go heteromeric G proteins. The involvement of Gi in mediating LPS effects is well established [31]. We also tested Caspase 3, an inflammatory marker in rodents [32] which is involved in the activation of a cascade of caspases to induce cell apoptosis. Specifically, Caspase 3 is involved in the cleavage of IL-1β to its mature form which induces a further inflammatory response. Finally, GM-CSF was shown to contribute to LPS-induced inflammation by augmenting the effects of pro-inflammatory cytokines. GM-CSF induces the priming of TNF-α and facilitates the differentiation of monocytes into macrophages through the ERK 1/2 signaling pathways [33,34]. Because these markers did not change significantly after treatment despite the significant alteration in spleen weight, we tested two cytokines further downstream, TNFα and IL-1β [35], which were previously shown to be involved in growth retardation [2,6,7]. However, there were no significant differences between the groups. This discrepancy may be due to the fact that we measured the mRNA levels and may have missed the immediate cytokine reaction to the LPS (which has a biological half-life of 7.5 h [22]), whereas the effect on the spleen weight was cumulative.

Both whey and soy are considered the best proteins in their categories because they contain all the essential amino acids, although in different relative amounts [36,37]. Proteins are not merely amino acid chains but can have additional functions. While LPS exposure increased the weight of the spleen, whey protected the animals from this effect, suggesting an anti-inflammatory function. Whey is known to consist of functional fractions which have multiple health benefits against infection and inflammation, such as glycomacropeptide, β-lactoglobulin, α-lactalbumin, and lactoferrin [38]. Ma et al. identified a specific peptide (DQWL) in whey which exerted an inhibitory activity on IL-1β and TNF-α mRNA expression in LPS-treated mouse macrophages in vitro [39]. This direct evidence for the in vitro anti-inflammatory effect of whey [39] supports the in vivo results presented in the current study.

The lesser weight gain in the whey-fed animals in the current study was also striking. Several studies have reported the presence of anti-obesity functional fractions in whey. These include α-lactalbumin [40] which suppresses hunger in humans, decreases weight gain and adiposity in rat and mice models, and improves glucose tolerance in diabetic rats [41,42]; lactoferrin [43], reported to modulate the gut microbiota and decrease weight gain [41]; and the hydrophilic glycopeptide, glycomacropeptide, that was shown to have beneficial effects on satiety [44]. We also reported this effect of whey on weight gain in a previous study, which may be important in circumventing the long-term complications of catch-up growth [20].

The beneficial effects of whey on bone growth may also stem from di-peptides that were identified as calcium chelating peptides involved in bone construction [26]. Whey is also especially rich in branched amino acids (BCAA) as well as sulfur-containing amino acids [45], including leucine, which is known to regulate protein synthesis [46], isoleucine, and valine, which all play crucial roles in metabolism, blood glucose homeostasis, neural function [47], and microbiome composition. Taken together, these qualities make whey a “functional food” that has health benefits beyond its nutritional value [48].

The results of this study clearly show that there were significant differences in the growth patterns and EGP height as a function of protein identity, even in the presence of inflammation. In our previous study, we compared whey to soy [21] in normal rats and showed that whey had a better effect on linear growth: the IGF-I levels were greater, the EGP was higher and better organized, the bone quality was improved, and weight gain was reduced [21]. In the current study, the EGP maintained its natural organization, suggesting conserved growth potential. However, we have no explanation as to why the differences in the EGP height were not immediately translated into differences in the humerus length. It is possible that a longer follow-up was required.

In the current study, we showed that most of the effect of both LPS and whey was concentrated on the HZ. There are several other cases in which the HZ of the EGP can be enlarged. For example, rats that were fed exclusively ultra-processed food exhibited an enlarged disorganized EGP [49]. Vegfa KO mice showed an enlarged HZ which lacked normal hypertrophic chondrocytes [50]. In rickets, the failure of cartilage to mineralize and undergo resorption is manifested by the loss of the columnar arrangement of chondrocytes, thickening and disorganization of the HZ with tongue-like projections of cartilage extending into the spongiosa, blurring of the limit between the PZ and HZ, and irregular penetration of blood vessels [51]. By contrast, in the current study, none of these phenomena were observed. The hypertrophic cells maintained their regular appearance, the columns were densely packed, and the boundaries of the EGP were intact and were not breached by either tongue-like projections of hypertrophic cells or vasculature.

Hypertrophic chondrocytes are considered the major factor regulating the growth rate in endochondral bones. Hypertrophic chondrocytes calcify the surrounding extracellular matrix and produce factors that attract bone cell precursors, bone cells, and blood vessel growth. The overall effect of this process of chondrocyte proliferation, hypertrophy, and ECM secretion is the elongation of bones and the progressive creation of new bone tissue at the bottom of the EGP. Given that the most visible effect of LPS on the EGP was in the HZ, it may be suggestive not only of possible alterations in growth potential but also of the risk of hindering bone quality in the critical timespan of linear growth. Because they are the last cells in the endochondral ossification process, hypertrophic chondrocytes can be considered the master regulators of future bone quality [52]. Indeed, in the above-mentioned meta-analysis, LPS negatively impacted the trabecular bone structure [12].

Our literature search identified only one other study that has investigated the effect of LPS on the EGP, which was conducted on chick embryos. The authors reported shorter limbs and a smaller EGP in the LPS-treated embryos compared to the controls. The relative HZ length was smaller [53], similar to our study. The authors reported that LPS exposure increased the expression of Sox9 and suggested that it could be partially responsible for delaying the chondrocyte transition from PZ to HZ [54]. In the current study, we found no differences in Sox9 expression, even though we measured Sox9 mRNA 3 days after the LPS treatment was discontinued. It is well-known that the mRNA and protein levels of SOX9 do not always match because the SOX9 protein outlives Sox9 mRNA in the upper hypertrophic chondrocytes [55]. However, the small amount of available biological material precluded us from examining the SOX9 protein levels. In addition to Sox9, we measured the expression of two hypertrophic markers: Coll X and MMP-13. Interestingly, they exhibited a different expression pattern over time, with significantly less Coll X expression and an increase in MMP-13 in the soy group but no such effects in the whey group. This was associated with the reduction in the height of the EGP and HZ, which was more pronounced in the soy group over time. The increase in MMP-13 may have been due to the residual effect of LPS on MMP-13 [56] that was corrected by whey but not by soy.

The IGF-I signaling pathway plays an important role in promoting hypertrophic chondrocyte formation [57]. These effects are predominantly due to local IGF-I receptors [58] whose expression is higher in the HZ [59]. Inflammation (i.e., LPS) causes a decrease in serum IGF-1 levels which may result in reduced hypertrophy in the EGP [57]. In previous studies conducted in our lab, we showed that rats fed with whey had a 20% increase in IGF-1 levels compared to rats fed with soy (whey, 1508 ± 94; soy, 1229 ± 93; *p* < 0.05) [21]. This may imply that whey can ameliorate the effect of inflammation by blocking the decrease in IGF-1 levels, therefore conserving the HZ in the EGP.

In our institute, an innovative nutritional formula consisting of whey was developed to support growth. The effect of this formula was tested in a prospective double-blind placebo-controlled trial in short and lean healthy children aged 3–9 years. The results showed that this nutritional supplement increased the height gain of the participants without an associated increment in their BMI [28,29]. The current study reproduced the same effect by demonstrating the positive and perhaps protective effect of whey on growth even in the presence of inflammation.

The results of this study are thus important for the development of therapeutic options in several inflammatory diseases causing growth attenuation, such as Crohn’s disease, cystic fibrosis, juvenile chronic kidney failure, and juvenile idiopathic arthritis.

## Figures and Tables

**Table 1:** Primers used in the study.

Gene	Forward Primer	Reverse Primer
TLR4	GCTGGGACTCTGATCATGGC	TCTGATCCATGCATTGGTAGG
CD14	GCCAGAGAACGCTGCTGTAA	AGGCATGCACCAGTGTCAAA
Caspase 3	ACGAACGGACCTGTGGACCTGAA	CCGGGTGCGGTAGAGTAAGCATA
GM-CSF	TGGCGCCTTGACCATGATAG	TTCACAGTCAGTTTCCGGGG
IL-1b	ATCTCACAGCAGCATCTCGA	ATCACACACTAGCAGGTCGT
TNFa	TCAAAACTCGAGTGACAAGCC	AGCCTTGTCCCTTGAAGAGA
Coll X	ACCCTGGTTCATGGGATGTTT	TATTGTGTCTTGGGGCTAGCAA
MMP-13	ATGAAGACCCCAACCCTAAGC	ATGGCATCAAGGGATAGGGC
SOX9	TGAAGAACGGACAAGCGGAG	ACTCATGCCGGAGGAGGAAT
GAPDH	GGTGCTGAGTATGTCGTGGA	CGGAGATGATGACCCTTTTG

## Data Availability

Data is contained within the article and Supplementary Material.

## Supplementary Materials

The following supporting information can be downloaded at: https://www.mdpi.com/article/10.3390/nu15081823/s1, Figure S1: Protein consumption was calculated per day as food consumption × 0.28 or 0.18 depending on the group (0.18 for CTL and LPS and 0.28 for the whey and soy groups). Daily *t*-tests between groups showed that there was no significant difference in daily protein consumption; Table S1: Macronutrient, vitamin, and mineral content of the experimental diets.
