# Supplementary material for: A Whey-Based Diet Can Ameliorate the Effects of LPS-Induced Growth Attenuation in Young Rats
Source: Nutrients. 2023 Apr 10;15(8):1823. doi: 10.3390/nu15081823 (PMC10146220; doi:10.3390/nu15081823)
Supplement: Supplementary file 1 [file nutrients-15-01823-s001.zip › nutrients-2298136-supplementary.pdf]

Supplementary Figure S1: Protein consumption was calculated per day as food consumption*0.28 or 0.18 depending on the group (0.18 for CTL and LPS and 0.28 for the whey and soy groups). Daily t-tests between groups showed that there was no significant difference in daily protein consumption.

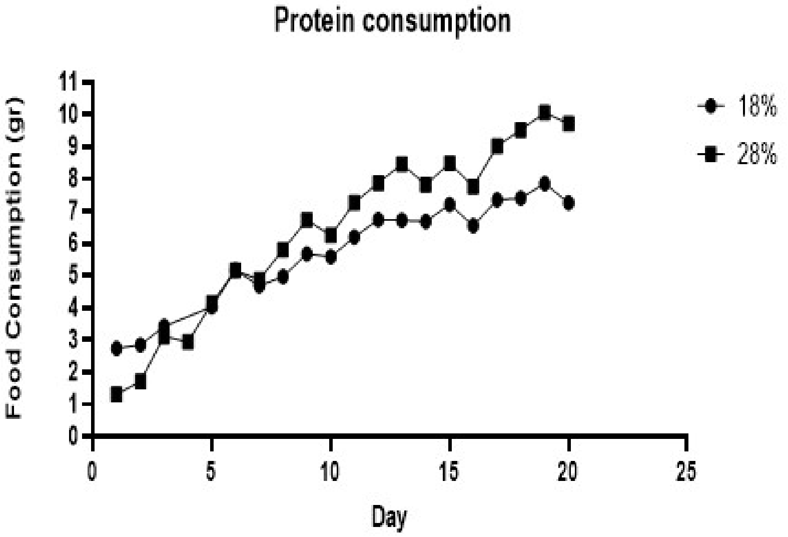

Supplementary Table S1. Macronutrient, vitamin, and mineral content of the experimental diets

	**TD190911 (whey)**	**TD190912 (soy)**
Energy (Kcal/g)	3.3	3.3
Carbohydrate (g/Kg)	383	395
Protein (g/Kg)		
Whey	232	
Soy		231
Fat (g/Kg)	91	93

Corn Starch (g/Kg)	260	271.207
Cellulose (g/Kg)	189.097	163.99
Minerals		
Calcium, g/kg	9.5	9.5
Phosphorus, g/kg	4.2	5.2
Sodium, g/kg	3.5	4.6
Potassium , g/kg	7.2	7.4
Chloride, g/kg	3.1	3.1
Magnesium, g/kg	1.0	1.2
Zinc, mg/kg	69.1	82.6
Manganese, mg/kg	20.5	25.0
Copper, mg/kg	11.7	15.1
Iodine, mg/kg	0.4	0.4
Iron, mg/kg	70.9	105.3
Selenium, mg/kg	0.29	0.29
Chromium, mg/kg	1.94	1.94
Vitamins		
A (IU/g)	15,000	15,000
B_1 (thiamin), mg/kg	17.8	17.8
B_2 (riboflavin), mg/kg	22.0	22.0
B_3 (niacin), mg/kg	99.0	99.0
$B_{6,}$ mg/kg	18.0	18.0

B_{12}, mg/kg	0.03	0.03
C, mg/kg	991	991
D, IU/g	1,500	1,500
E, mg/kg	100	100
Choline, mg/kg	1,216	1,216
Biotin, mg/kg	0.4	0.4
Folic acid, mg/kg	2	2
Inositol, mg/kg	110	110
K_3, mg/kg	50	50
Pantothenic acid	60	60
